# The long-term course of subsolid nodules and predictors of interval growth on chest CT: a systematic review and meta-analysis

**DOI:** 10.1007/s00330-022-09138-y

**Published:** 2022-09-22

**Authors:** Linyu Wu, Chen Gao, Ning Kong, Xinjing Lou, Maosheng Xu

**Affiliations:** 1 Department of Radiology, The First Affiliated Hospital of Zhejiang Chinese Medical University (Zhejiang Provincial Hospital of Traditional Chinese Medicine), 54 Youdian Road, Hangzhou, China; 2 The First School of Clinical Medicine of Zhejiang Chinese Medical University, Hangzhou, China

**Keywords:** Lung neoplasms, Tomography, x-ray computed, Follow-up studies, Risk factors, Meta-analysis

## Abstract

**Objectives:**

To calculate the pooled incidence of interval growth after long-term follow-up and identify predictors of interval growth in subsolid nodules (SSNs) on chest CT.

**Methods:**

A search of MEDLINE (PubMed), Cochrane Library, Web of Science Core Collection, and Embase was performed on November 08, 2021, for relevant studies. Patient information, CT scanner, and SSN follow-up information were extracted from each included study. A random-effects model was applied along with subgroup and meta-regression analyses. Study quality was assessed by the Newcastle–Ottawa scale, and publication bias was assessed by Egger’s test.

**Results:**

Of the 6802 retrieved articles, 16 articles were included and analyzed, providing a total of 2898 available SSNs. The pooled incidence of growth in the 2898 SSNs was 22% (95% confidence interval [CI], 15–29%). The pooled incidence of growth in the subgroup analysis of pure ground-glass nodules was 26% (95% CI: 12–39%). The incidence of SSN growth after 2 or more years of stability was only 5% (95% CI: 3–7%). An initially large SSN size was found to be the most frequent risk factor affecting the incidence of SSN growth and the time of growth.

**Conclusions:**

The pooled incidence of SSN growth was as high as 22%, with a 26% incidence reported for pure ground-glass nodules. Although the incidence of growth was only 5% after 2 or more years of stability, long-term follow-up is needed in certain cases. Moreover, the initial size of the SSN was the most frequent risk factor for growth.

**Key Points:**

*• Based on a meta-analysis of 2898 available subsolid nodules in the literature, the pooled incidence of growth was 22% for all subsolid nodules and 26% for pure ground-glass nodules.*

*• After 2 or more years of stability on follow-up CT, the pooled incidence of subsolid nodule growth was only 5%.*

*• Given the incidence of subsolid nodule growth, management of these lesions with long-term follow-up is preferred.*

**Supplementary Information:**

The online version contains supplementary material available at 10.1007/s00330-022-09138-y.

## Introduction

Subsolid nodules (SSNs), sometimes named ground-glass nodules, can be categorized as pure ground-glass nodules (pGGNs) and mixed ground-glass nodules (mGGNs) [1]. According to the guidelines of the National Comprehensive Cancer Network and the Fleischner Society for the management of SSNs, thoracic CT should be conducted every 6–12 months for solitary pGGNs (6 mm or larger) or every 3–6 months for mGGNs (6 mm or larger) and multiple SSNs to determine if the nodules are persistent [1, 2]. Although the growth of SSNs is indolent, the probability of malignancy in persistent SSNs is higher than that of solid nodules [3].

Persistent SSNs usually consist of atypical adenomatous hyperplasia, adenocarcinoma in situ, minimally invasive adenocarcinoma, or invasive adenocarcinoma [4, 5]. Because of the indolent biological behavior of adenocarcinoma in situ, it was reclassified as a precursor glandular lesion in 2021 and was found to not require surgery [4]. Numerous reports have indicated that long-term follow-up CT (e.g., for at least 5 years) is recommended for SSNs because of their indolent clinical course [1, 3, 6–10]. If the SSN grows or develops a solid component, surgery should be considered because of the higher risk for invasive adenocarcinoma in these nodules [7–10]. Up to a 10% solitary pGGN growth rate has been reported, even in SSNs measuring 5 mm or smaller, with a long-term follow-up of at least 5 years [11]. Lee JH et al found that only 2/235 (2%) SSNs measuring 6 mm or larger after 5 years of stability showed subsequent growth [9]. However, to our surprise, Lee HW et al found that subsequent SSN growth was identified in 27/208 (13.0%) that had been stable for 5 years [3]. Therefore, the long-term natural course of SSNs is still unclear.

To our knowledge, the pooled incidence of interval growth after long-term follow-up has not yet been systematically evaluated. Thus, the purpose of this systematic review and meta-analysis was to estimate the incidence of interval growth after long-term follow-up and identify the predictors of interval growth in SSNs on chest CT. We also calculated the pooled growth incidence of SSNs after at least 2 years of stability.

## Materials and methods

This systematic review and meta-analysis were conducted and reported according to the Preferred Reporting Items for Systematic Reviews and Meta-Analysis (PRISMA) guidelines [12]. This study was exempt from ethical approval at our institution. The review was registered on PROSPERO before initiation (registration no. CRD42021293524).

### Search strategy

A comprehensive search of MEDLINE (PubMed), Cochrane Library, Web of Science Core Collection, and Embase was performed on November 08, 2021, to identify studies reporting the growth of SSNs. The search terms were as follows: (“ground-glass nodule*” OR “subsolid nodule*” OR “part-solid nodule*” OR “lung nodule*”) AND (“growth” OR “nature course” OR “natural history” OR “follow up”) AND (“computed tomography” OR “CT”). The detailed search strategy is described in the Supplementary Materials. Only original articles were considered for analysis, and there was no limit on the year or language of publication.

### Eligibility criteria

The first selection was performed by two independent readers with 8 years and 5 years of experience in thoracic imaging (L.W. and C.G., respectively). First, all the articles obtained from the above four databases were combined, and then duplicate articles were removed. Second, the relevant articles were screened by their titles and abstracts. Finally, the relevant articles were reevaluated through full-text retrieval to find eligible articles.

Articles that reported the growth of SSNs after follow-up and/or predictors of interval growth were included. These included studies in which SSNs were followed up for two or more years and studies that followed up the SSNs for less than 2 years but reported SSN growth ≥ 2 years of stability. The following articles were excluded: (1) case reports, conference abstracts, comments, editorials, letters to the editor, and guidelines; (2) studies based on all types of nodules and those that did not specifically mention SSNs; (3) articles with missing data or overlapping patients; (4) studies in which the duration of follow-up was less than 2 years or unknown and those that did not report SSN growth after ≥ 2 years of stability; and (5) studies in which all patients had a history of malignant tumors or residual SSNs after surgical treatment of the dominant lung cancer.

### Data extraction

For each analyzed article, the recorded data included first author; country; year of publication; study design; CT scanner; tube voltage or tube current; reconstruction slice thickness; window width, window level; plain or enhanced CT; reconstruction algorithm; number of patients and nodules; patient age; number of pGGNs and mGGNs; baseline size of the SSNs; nodule measurements; follow-up period; definition of growth; definition of SSN; number of growths; number of growths after ≥ 2 years of stability; growth patterns; pathological diagnosis; interval between detection and interval growth; odds ratio and 95% confidence interval (CI) in multivariate analysis for growth; and hazard ratio in Cox analysis for the time of the growth.

The purpose of this study was to calculate the pooled incidence of interval growth after long-term follow-up and find the predictors of interval growth in SSNs on chest CT. Descriptive statistics were used to summarize the characteristics, growth incidence, and risk factors for SSNs. The incidence of interval growth after long-term follow-up was pooled. Subgroup analysis was conducted separately for pGGNs and mGGNs. If a sufficient amount of homogeneous data were available, the pooled incidence of interval growth after at least 2 years of stability was calculated. Another subgroup analysis was conducted separately for subsolid nodule growth after 2 years of stability or more for SSNs ≥ 5 mm and < 5 mm.

### Statistical analysis

A random-effects model was used to calculate the pooled incidence of growth and its 95% CI. Heterogeneity between the studies was assessed using both Q and *I*^*2*^ statistics. The heterogeneity was considered high if *I*^*2*^ was greater than 50%, and high heterogeneity may affect the extent to which generalizable conclusions can be formed [13, 14]. Analysis was conducted using Stata 16 software (StataCorp) and R software version 4.2.1 (https://www.r-project.org/). The methodological quality of the observational studies included in the review was appraised with the Newcastle–Ottawa Scale (NOS) [15]. Study quality was evaluated by NOS scores, and divided into high (score of 7–9), moderate (score of 4–6), and poor (score of 0–3) [16]. The Egger test was used to assess publication bias. Finally, *p *< .05 was therefore considered to indicate a significant difference.

## Results

### Literature search

**Fig. 1:**
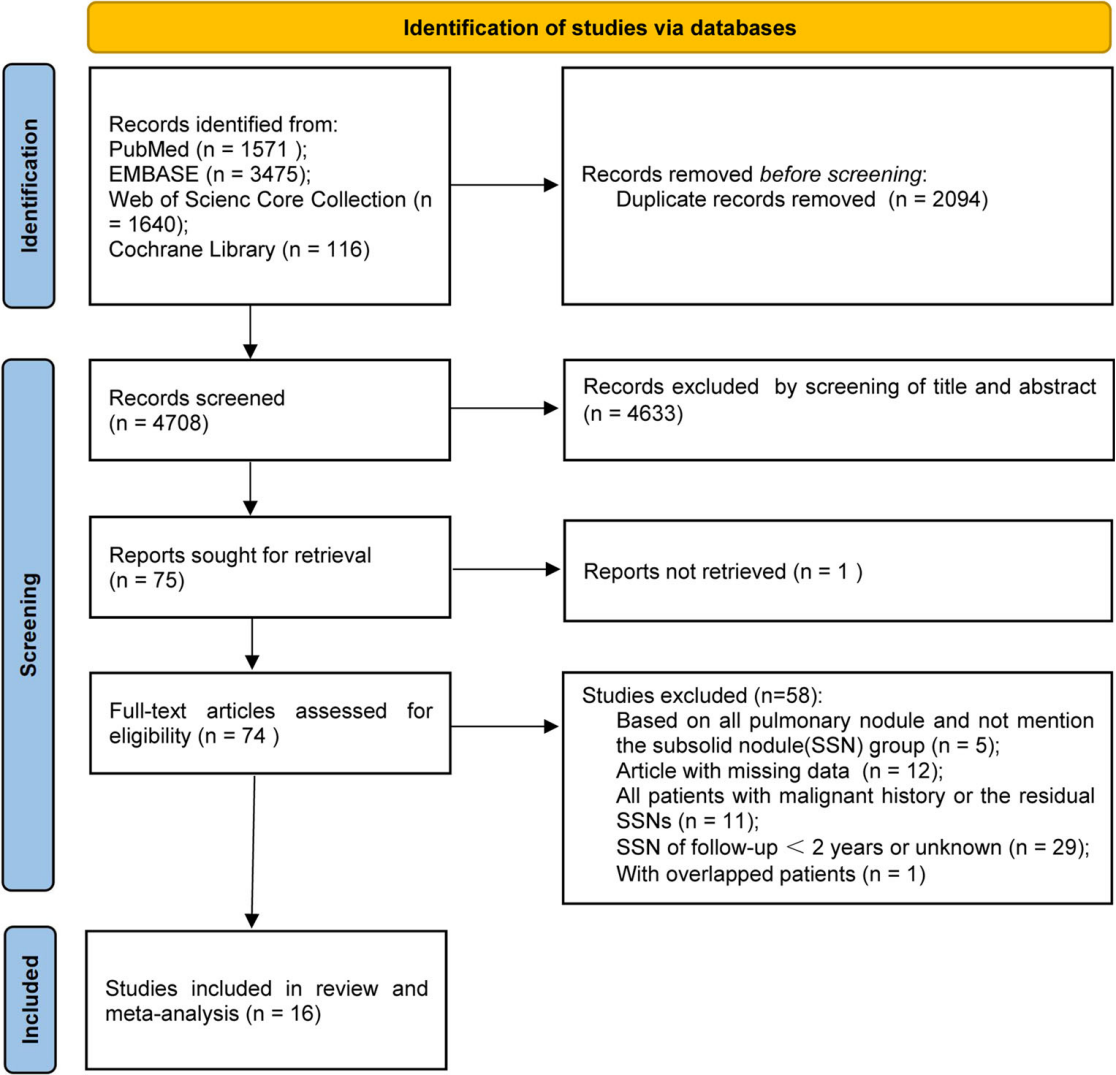
Flowchart of the literature search for this systematic review and meta-analysis

The literature search flow diagram is shown in Fig. 1. A total of 16 articles from 6802 initially retrieved articles were included in the study after strict application of the inclusion and exclusion criteria [3, 7–11, 17–26]. The detailed criteria of the terms for SSN follow-up in the included studies were provided in Supplementary Materials, Table E1. The 16 included articles, with a total of 2898 available SSNs, were published from 2012 [17] to 2020 [9].

### Study characteristics

The main characteristics of the analyzed studies are shown in Tables 1, 2, 3, and 4. CT scanner information of the included studies is shown in Table 1. All the study patients were from Asia (6 Japan, 4 China, 6 Korea). Only one study was prospective [11], and the other studies were retrospective [3, 7–10, 17–26]. The reconstruction slice thickness ranged from 0.625 to 5 mm. Of the studies that reported the reconstruction slice thickness, thirteen of 14 (92.86%) reported values of 3 mm or less [3, 7, 8, 10, 11, 18, 20–26], while only one (7.14%) reported values of 1 to 5 mm [9]. In total, 12/14 (85.71%) studies used two or more CT scanners [3, 7, 9–11, 17, 18, 20, 21, 23, 24, 26].

**Table 1:** CT scanner information of the included studies

Study	Country^*^	Study design	CT scanner	Tube voltage/ Tube current	Reconstruction slice thickness (mm)	Window width, window level (HU)^#^	Plain or enhanced CT	Reconstruction algorithm
Takahashi et al, 2012 [17]	Japan	R	four-detector row scanners; 64-detector row scanner	140 kVp; 160–250 mAs	--	1500 or 1750, -600 or -700	Plain CT	High-spatial-frequency algorithm
Chang et al, 2013 [18]	Korea	R	64-detector row scanners; single-detector scanner	120 kVp; 40 mA	< 2.5	1500, -700	Low-dose plain CT	High-spatial-frequency algorithm
Kobayashi et al, 2013 [19]	Japan	R	--	--	--	1000 to 2000, -500 to -700	--	--
Lee et al, 2013 [20]	Korea	R	the Brilliance-64, MX-8000 IDT; iCT 256	--	1 or 3	1500, -600	--	--
Eguchi et al, 2014 [21]	Japan	R	Hispeed Advantage RP, Light Speed Ultra; Light Speed VCT Vision	--	1.25	1500, -550	Plain CT	--
SHIN et al, 2014 [22]	Korea	R	64-detector row scanners	120 kVp; 20 mA	1.25	--	--	--
Kakinuma et al, 2015 [11]	Japan	P	16-row scanner; 64-row CT scanner	120 kVp; 30 mA	1 or 2	2000, -600	--	Standard reconstruction kernel (function kernel 01 or 03)
Cho et al, 2016 [7]	Korea	R	the Brilliance-64, MX-8000 IDT,and iCT 256	--	1 to 3	1500, -600	--	--
Sawada et al, 2016 [8]	Japan	R	--	--	1	--	Plain CT	--
SATO et al, 2017 [10]	Japan	R	Light Speed VCT or Optima CT 660	--	0.625 to 2	1500, -650	--	--
Lee et al, 2019 [3]	Korea	R	16-slice detector CT scanner; 256-slice multidetector CT scanner	120 kVp, 30 mAs or 120 kVp, 20 mAs	1 or 3	1500, -600	Low-dose CT	--
Qi et al, 2019 [23]	China	R	64-detector row scanners (LightSpeed VCT, Discovery CT750 HD or Optima CT660, General Electric Medical Systems; TOSHIBA Aquilion, TOSHIBA Medical Systems)	120 kVp,35 mAs or 120 kVp, 200–350 mAs	1 or 1.25	1600, -600	Low dose plain CT/ enhanced CT	Standard reconstruction algorithm
Shi et al, 2019 [24]	China	R	Siemens, Somatom Definition AS scanner; Philips, Brilliance 40 scanner	120 KVp; 200 mAs	1	1400, -450	Plain CT	Standard soft kernel (Siemens B31 filter); sharp reconstruction kernel
Gao et al, 2020 [25]	China	R	Siemens Somatom sensation, 64 slice, CT scanner	120 kVp; automatic tube currentmodulation	0.75	--	Plain CT	Reconstruction kernel, B31f
Qiu et al, 2020 [26]	China	R	Discovery HD750-64; LightSpeed VCT; Somatom Definition Sensation-64; Somatom Definition Flash Sensation-64	100–120 kVp, 200–280 mAs	0.625 to 1	1200, -600	--	--
Lee et al, 2020 [9]	Korea	R	Brilliance 64, Ingenuity, ICT 256, Mx 8000, Phillips Medical Systems; Sensation 16, Somatom Definition, Siemens Medical Solutions; Aquilion One, Toshiba; Discovery CT 750 HD, Light Speed Ultra, GE Medical Systems	--	1 to 5	--	Plain CT/ enhanced CT	--

Note: *R*, retrospective; *P*, prospective; “‐‐”: not mentioned; *: country was based on the corresponding author; “#”: the lung window setting

**Table 2:** Initial patient/nodule characteristics and long-term follow-up of subsolid nodules in the included studies

Study	No. Patients/nodules	Mean /median patient age(years)	Gender (female/patients)	No. of SSNs (pGGN;mGGN)	Baseline Size of SSN(mm)；mean ± SD, media (range)	Measurement of nodule (LD/MD)	Follow-up Period (mean, median, (range)), years	Definition of growth	Definition of SSN	No. of growth	No. of growth after ≥ 2 years’ stability)	Growth patterns	SSN confirmed by pathology	Interval between detection and interval growth (months) (mean ± SD, media (range))	Note
Takahashi et al, 2012 [17]	111/150	Mean 62.6 ± 10.5 (33–85)	77/111	150 pGGN	Mean, 8.1 ± 2.4	LD	Mean, 5.5 ± 2.08 (2.89–9.46)	D^$^	VA	19 /150	6 / 150 (≥ 2 years stability)	pGGN: D (*n* = 15); D + NS (*n* = 4)	3 adenocarcinomas; 5 BAC	Mean 24.16	pGGN ≤ 15mm, FU ≥ 2.5 years
Chang et al, 2013 [18]	89/122	Median, 53 (37–70)	16/89	122 pGGN	Median, 5.5 (3–20)	LD	Median, 4.92 (2.08 – 11.67)	D	TDR	12 / 122	--	pGGN: D (*n* = 12)	11 primary lung cancer	--	pGGN, FU > 2 years
Kobayashi et al, 2013 [19]	61/108	Median, 61 (35–78)	39/61	82 pGGN; 26 mGGN	median, 9.5 (4–25)	LD	median 4.2	D	VA	29 / 108	4 / 108 (≥ 2 years stability)	SSN: D (*n* = 15); mGGN: D+ DS (*n* = 14)	5 IAC; 9 MIA; 11AIS; 1 AAH	--	ground glass opacity proportion of 50% or more
Lee et al, 2013 [20]	114/175	61(37–92)	45^&&^/114	143 pGGN; 32 mGGN	Mean, 7.8 ± 4.4 (range, 2.5–31.0)	LD	Median, 4^&&^ (2–8.25)	D	VA	46^&&^ / 175	2 / 90 (≥ 4 years stability)	pGGN:D (*n* = 17)；D + NS (*n* = 9); NS (*n* = 2); mGGN: D (*n* = 15)；D + DS^^^(*n* = 3)	11IAC;11MIA; 3AIS; 1AAH; 1 pleomorphic carcinoma; 2 interstitial fibrosis	Mean, 48.8 ± 19.4	SSN, FU > 2 years
Eguchi et al, 2014 [21]	124/124	Mean 64.5 ± 10.4	87/124	124 pGGN	Mean, 7.4 ± 2.8	MD	Median, 4.75 (2.01 – 9.47)	D; NS	VA	64/124	--	pGGN: NS (*n* = 40), D (*n* = 24)	5AIS; 15MIA; 12 IAC; 1 pulmonary capillary hemangiomatosis with foci	Median, 38.0 (3.1 - 80.0)	pGGN，FU > 2 years
SHIN et al, 2014 [22]	--/70	--	--	70 SSN	Mean, 7 ± 1.2; median, 7 (4–9)	LD^#^	≥ 2	V	--	13/70	2/70 (≥ 2 years stability)	--	4 IAC; 1 MIA; 1 squamous cell carcinoma	--	3mm ≤ SSN < 10 mm, FU ≥ 5 years or diagnosed of cancer within a 5-year period
Kakinuma et al, 2015 [11]	439/439	Mean 58.9，median 59.0 (40–78)	192/439	439 pGGN	Mean, 3.9; median, 4 (1.5–6.5)	MD	Median, 6 (2.4–9.1)	D	VA	45/439	--	--	1AAH; 2MIA; 2IAC	--	Solitary pGGN ≤ 5mm (437 FU ≥ 5 years)
Cho et al, 2016 [7]	218/453	Median 56 (21–86)	110/218	438 pGGN;15 mGGN	Median 5.0 (2.0–31.1)	LD	Median 6.46 (3.18–9.76)	D; DS; NS； NS + De	TDR	15/453	15/453 (≥ 3 years stability)	pGGN : D + NS (*n* = 1); NS + De (*n* = 1); mGGN: D + DS (*n* = 1); D (*n* = 12)	5IAC; 2MIA	--	FU > 3 years stability
Sawada et al, 2016 [8]	226/226	Median 61 (20–82)	148/226	166 pGGN; 60 mGGN^&&^	Median 10 (3–30)	--	––	Increase in tumor size/ CTR	CTR	39/226*	11/226 (≥ 2 years stability)	--	63AIS; 36MIA; 25IAC	Median 24 (3–108)	
SATO et al, 2017 [10]	187/187	Mean 65.5 ± 11.6	118/187	134 pGGN;53 mGGN	Mean 12.2 ± 6.1	LD	Median, 3.7(2.01 – 7.25)	D; DS; NS	VA	62/187	13/187 (≥ 3 years stability)	--	25IAC; 5AAH/AIS/MIA	--	Ground-glass opacity ≥ 50%, FU > 2 year
Lee et al, 2019 [3]	160/208	Median 52 (28–84)	68/160	162 pGGN; 46 mGGN	Median 4.7 (1.7–10.0)	LD	median, 11.3(10 – 14.92)	D; D+DS/ NS**	--	27 / 208 (≥ 5 years stable)	27 / 208 (≥ 5years stability)	D + NS (*n* = 16); D (*n* = 11)	1AIS; 1MIA; 1IAC	median 103 (60–141)	FU ≥ 5 years stability
Qi et al, 2019 [23]	110/110	Mean 54.3 ± 9.7	72/110	110 pGGN	Mean 8.7 ± 3.2	MD	Mean, 4.06 ± 1.98	V; NS	--	52 / 110	25/110(≥ 2 years stability)	pGGN: V (*n* = 52)	1 Focal fibrosis; 2AAH; 1AIS; 3MIA; 23IAC; 1unknown	28.47 ± 22.5 (3.87–95.20)	pGGN (99 FU ≥ 2 years, and 11 FU < 2 years but showed growth)
Shi et al, 2019 [24]	59/101	Median, 61(40–85)	19^&&^/59	101 pGGN	--	LD	Median, 4.33 (2.67–5.75)	Quantitative analysis: 3D; V/S; radiological assessment: D; NS	--	16/101	--	pGGN: D (*n* = 7); NS (*n* = 3); 3D /V/M (*n* = 6)	--	--	5 mm ≤ pGGN ≤ 3 0 mm；FU > 2 years
Gao et al, 2020 [25]	85/110	Training set: mean, 56.8 ± 11.9; validation set: mean, 59.2 ± 16.2	59/85(78/110^&^)	83 pGGN; 27 mGGN	Training set: mean, 8.1 ± 3.8; Validation set: mean, 8.5 ± 2.9	LD	≥ 2	D; DS; NS	--	36/110	--	--	--	--	5mm ≤ SSN ≤ 30mm; FU ≥ 2 years
Qiu et al, 2020 [26]	75/80	Stable group:60 ± 11; growth group: 66 ± 10	--^&&^	80 pGGN	Stable group: mean, 7 ± 1; growth group: mean, 11 ± 3.5	MD	≥ 3	D; NS	VA	29/80	--	pGGNs: D (*n* = 25); NS (*n* = 4)	7 IAC; 3 AAH/AIS	--	pGGN ≤ 20mm，FU ≥ 3years
Lee et al, 2020 [9]	235/235	Mean, 64 ± 10	132/235	211 pGGN; 24 mGGN	Mean 8 ± 2 (6–17)	MD	Median, 9.33(7–17.33)	D; DS; NS	--	5/235	5/235(≥ 5 years stability)	pGGN: D (*n* = 2), NS (*n* = 1); mGGN: DS (*n* = 2)	1AAH; 1AIS; 5IAC	Median, 99 (84-146)	SSN ≥ 6mm，FU ≥ 5 years stability, FU ≥ 7years；age ≥ 35years

Note: “‐‐”: not mentioned; *FU*, follow-up; *SSN*, subsolid nodule; *pGGN*, pure ground-glass nodule; *mGGN*, mixed ground-glass nodule; *SD*, standard deviation; *D*, increase in mean/longest diameter of 2 mm or more; *DS*, solid portion increase of 2 mm or more; *NS*, new occurrence of solid part; *3D*, increase in 3D diameter of 2 mm or more; *V*, increase in volume by at least 20% or 25%; *V/*S, an increase of at least 30% in volume or mass; *NS + De*, NS + decreased ≥ 2mm in the whole size; *LD* (the longest diameter), the longest diameter on transverse CT sections and lung window setting; MD (mean diameter), the average of its maximal length and maximal orthogonal diameter on transverse CT sections and lung window setting; *AAH*, atypical adenomatous hyperplasia; *AIS*, adenocarcinoma in situ, *MIA*, minimally invasive adenocarcinoma; *IAC*, invasive adenocarcinoma; *BAC*, bronchi-alveolar carcinoma; *VA*, visual assessment; *CTR*, the ratio of the maximum diameter of consolidation relative to the maximum tumor diameter from the lung window; *TDR*, tumor shadow disappearance rate

*: included those follow up < 2 years

$: including those apparent visual change of the nodular area because of a change of the shortest nodule diameters

^: mGGN increased significantly in size and became solid masses

#: the maximum diameter

**: increase in solid portion in mGGN or new occurrence of solid part in pGGN

&&: inconsistency of the data in the article

&: based on nodules

**Table 3:** Predictive factors for subsolid nodule growth by multivariate analysis

Study	number of risk factors	multivariate analysis for growth	OR (95% CI)
Lee et al, 2013 [20]	3	Initial size ≥ 10 mm	6.46 (2.69–15.6)
		Presence of a solid portion	2.69 (1.11–6.95)
		Age ≥ 65 years	2.55 (1.13–5.77)
Eguchi et al, 2014 [21]	2	Smoking history	0.189 (0.056–0.635)
		Mean CT attenuation value	0.985 (0.979–0.990)
Cho et al, 2016 [7]	5	Age ≥ 65 years	5.51 (1.46–20.90)
		History of lung cancer	6.44 (1.73–24.00)
		Initial size ≥ 8 mm	5.74 (1.58–20.92)
		Presence of a solid portion	16.58 (2.04–134.70)
		Air Bronchogram	5.83 (1.41–24.19)
SATO et al, 2017 [10]	2	Past history of lung cancer	5.22 (1.38–23.8)
		GGN size ≥ 10 mm	43.6 (6.01–998)
Shi et al, 2019 [24]	2	Larger 3D maximum diameter	0.896 (0.820–0.948)
		Higher standard deviation	0.810 (0.723–0.883)
Gao et al, 2020 [25]	2	Diameter	1.087 (0.785–1.564)
		Rad-score	5.130 (0.948–37.835)

Note:*GGN*, ground-glass nodule; *OR*, odds ratio; *95% CI*, 95% confidence interval

**Table 4:** Predictive factors for the time to subsolid nodules growth by multivariate Cox analysis

Study	number of risk factors	Cox analysis for the time to growth	HR (95% CI)
Eguchi et al, 2014 [21]	4	Smoking history	2.388 (1.348–4.229)
		Tumor size ≥ 7 mm	2.336 (1.361–4.012)
		Mean CT attenuation value ≥ −670 HU	5.933 (3.237–10.873)
		With multiple GGNs	1.800 (1.039–3.119)
SATO et al, 2017 [10]	1	GGN size ≥ 10mm	23.3 (4.82–418)
Lee et al, 2019 [3]	3	Bubble lucency	12.455 (2.910–53.306)
		History of cancer other than lung cancer	3.140 (1.079–9.139)
		Development of a new solid component	19.140 (7.490–48.911)
Qi et al, 2019 [23]	4	Lobulated sign	0.504 (0.259–0.981)
		Initial mean diameter	1.438 (1.211–1.708)
		Initial volume	0.998 (0.996–0.999)
		Initial mass	1.006 (1.001–1.011)
Shi et al, 2019 [24]	2	The 3D maximum diameter	3.75 (2.14–6.55)
		Standard deviation	2.06 (1.35–3.14)
Qiu et al, 2020 [26]	2	The size of the lesion	9.18 (2.23–37.85)
		Blood vessel types (Type I)	0.22 (0.06–0.81)

Note:*GGN*, ground-glass nodule; *HR*, hazard ratio; *95% CI*, 95 % confidence interval

Type I: “intact vessels passing by or going through pure ground-glass nodule without tiny branches”

Except for one article that was based on all pulmonary nodules but reported an SSN subgroup [22], the other articles were all based only on SSNs [3, 7–11, 17–21, 23–26] (Table 2). There were 2545 pGGNs, 283 mGGNs [3, 7–11, 17–21, 23–26], and 70 SSNs that were not classified [22] (Table 2). Fourteen studies reported the sex of the patients with SSNs [3, 7–11, 17–21, 23–25], and 1182/2218 (53.29%) were female. The age of the patients ranged from 20 to 92 years [3, 7, 8, 11, 17–20, 24].

### Definition of growth and growth patterns

The definitions of SSN growth in the studies were as follows: 2 mm or more increase in mean/longest diameter [3, 7, 9–11, 17–21, 24–26]; 2 mm or more increase in the solid portion [3, 7, 9, 10, 25]; new occurrence of solid parts [3, 7, 9, 10, 21, 23–26]; 2 mm or more increase in the 3D diameter [24]; an increase of at least 30% in volume or mass [24]; increase in volume by at least 20% [22, 23]; new occurrence of a solid part and ≥ 2 mm decrease in overall size [7]; and increase in tumor size/the ratio of the maximum diameter of the consolidation relative to the maximum tumor diameter in the lung window [8]. The growth patterns of the included SSNs are shown in Table 2.

### Overall incidence of SSN growth

**Fig. 2:**
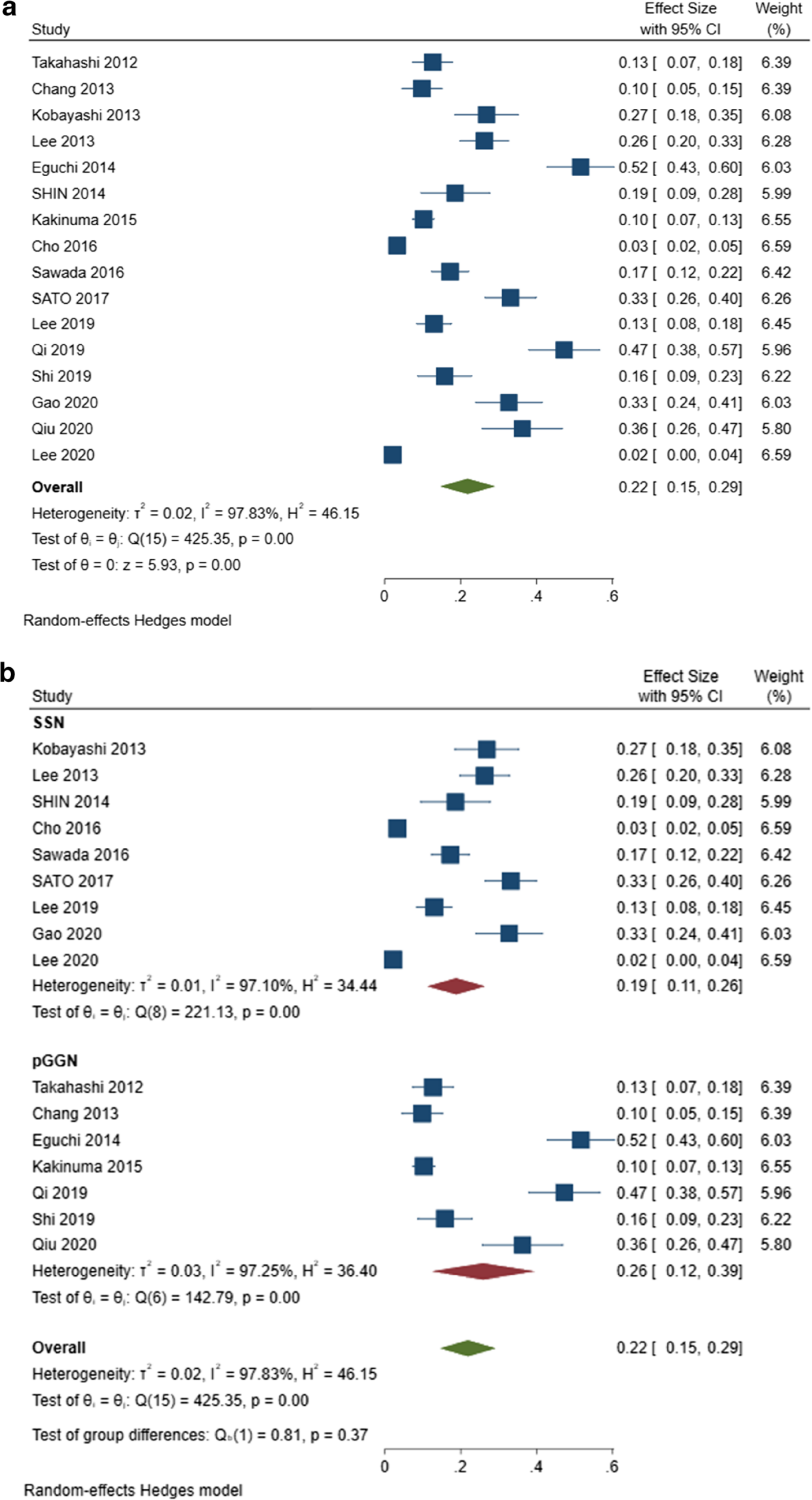
Forest plot of the overall incidence of subsolid nodule growth (**a**) and forest plot of incidence of growth among the pure ground-glass nodules and remaining subsolid nodule subgroups (**b**)

The pooled overall incidence of growth in all included studies was 22% (95% CI, 15–29%) (Fig. 2). In the subgroup analysis, the pooled incidence of pGGN growth was 26% (95% CI: 12–39%). The remaining SSNs, minus the pure ground-glass nodules were included in a subgroup of the remaining SSNs. The pooled incidence of growth was not different between pGGNs (26%, 95% CI: 12–39%) and the remaining SSNs (19%, 95% CI: 11–26%) (*p* = 0.37) (Fig. 2). High heterogeneity was found among the studies in the overall incidence of growth in SSNs (Q = 425.35, *p* < 0.001, *I*^*2*^ = 97.83%), pGGNs (Q =142.79, *p* < 0.001, *I*^*2*^ = 97.25%), and remaining SSNs (Q = 221.13, *p* < 0.001, *I*^*2*^ = 97.10%) (Fig. 2).

### Incidence of growth of SSNs after ≥ 2 years of stability

**Fig. 3:**
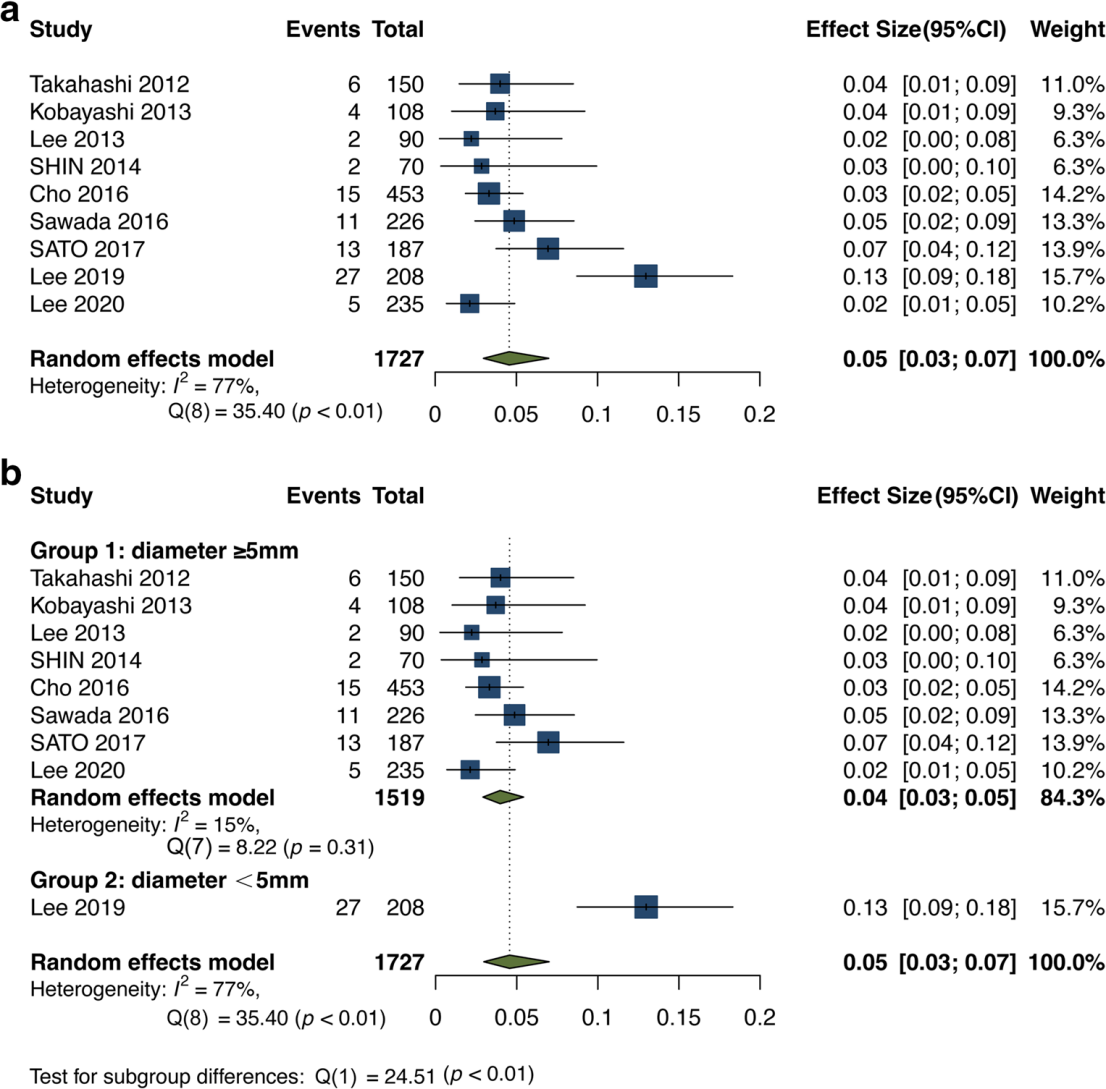
Forest plot of the incidence of subsolid nodule growth after 2 years of stability or more (**a**) and forest plot of the incidence of subsolid nodule growth after 2 years of stability or more for the subgroup analysis for SSNs ≥ 5 mm and < 5 mm (**b**). Diameter: the initial mean/median diameter of the SSNs

**Fig. 4:**
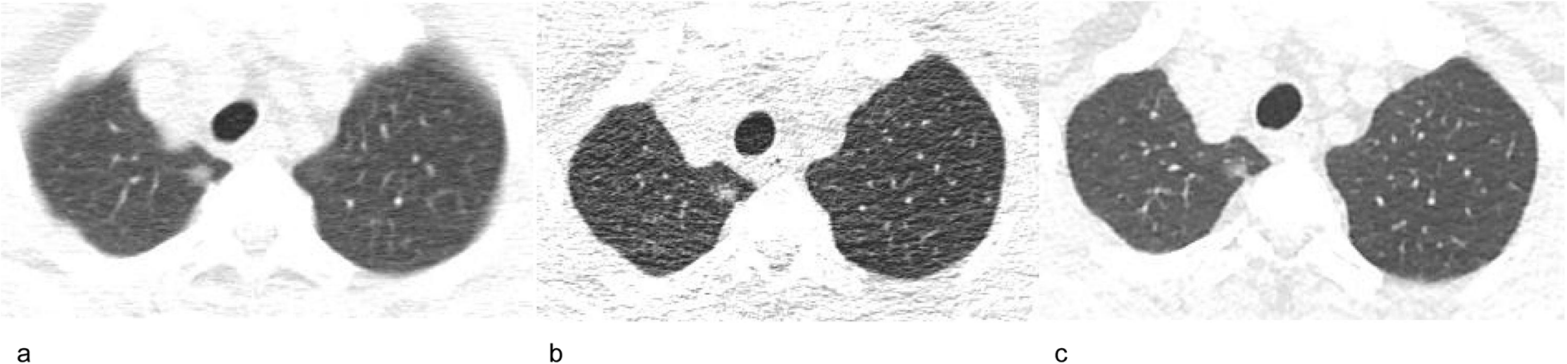
A 70-year-old man with a stable subsolid nodule after long-term follow-up CT. **a** Transverse plain CT section of a part-solid nodule in the right upper lobe. The nodule size (longest diameter) was 9 mm on transverse CT images at baseline. **b** Follow-up CT obtained 5 years after baseline showed that the nodule is stable. **c** The nodule was still stable after a 10-year follow-up

**Fig. 5:**
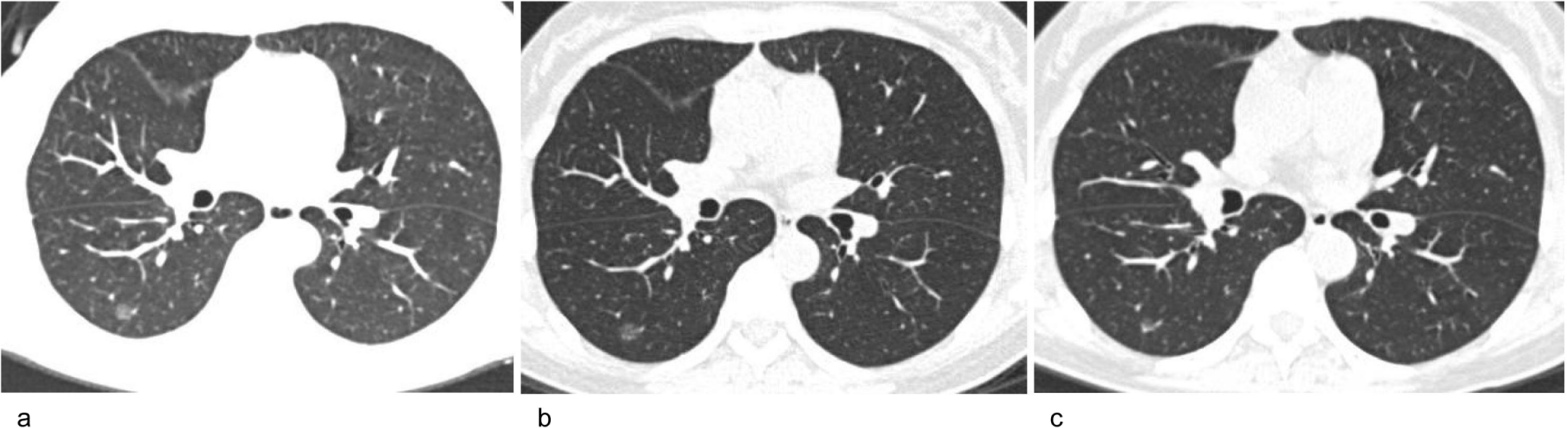
A 49-year-old woman with subsolid nodule growth after long-term follow-up CT. **a** Baseline CT. Transverse plain CT of a pure ground-glass nodule in the right lower lobe. The nodule size (longest diameter) was 8 mm on transverse CT images. **b** Follow-up CT obtained 5 years after baseline. The pure ground-glass nodule was stable. **c** Follow-up CT obtained 6 years after the baseline CT. A new solid component appeared in the nodule, and its size decreased from 8 to 6 mm. The growing nodule was a minimally invasive adenocarcinoma, as confirmed by pathology

After 2 or more years of stability (ranging from 2 to 5 years), the incidence of SSN growth was only 5% (95% CI: 3–7%) [3, 7–10, 17, 19, 20, 22]. The heterogeneity of this analysis was lower than that of the overall analysis (Q = 35.40, *p* < 0.01, *I*^*2*^ = 77.00% vs. Q = 425.35, *p* < 0.001, *I*^*2*^ = 97.83%) (Figs. 2 and 3). Another subgroup analysis based on the initial mean/median diameter of SSNs was conducted (Table E2 and Fig. 3b). When we removed the study with an initial mean/median diameter < 5 mm [3] for subgroup analysis, there was no heterogeneity in the subsequent analysis (Q = 8.22, *p* = 0.31, *I*^*2*^ = 15.00%). The incidence of growth after 2 years of stability or more for SSNs with an initial diameter ≥ 5 mm was 4% (95% CI: 3–5%) (Fig. 3b). Patient examples of stable and growing SSNs after long-term follow-up are shown in Figs. 4 and 5, respectively.

### SSNs confirmed by pathology

A total of 14 studies [3, 7–11, 17–23, 26] reported that some SSNs were confirmed by surgery or biopsy after long-term follow-up. Of these 329 SSNs, only 4/329 (1.2%) were benign (3 interstitial fibrosis; 1 pulmonary capillary hemangiomatosis with foci). A total of 325/329 (98.8%) SSNs were pathologically proven to be lung cancers or precursor glandular lesions. A total of 307/329 (93.3%) SSNs were lung adenocarcinomas or precursor glandular lesions, two SSNs were pleomorphic carcinoma or squamous cell carcinoma, five SSNs were bronchi-alveolar carcinomas and the other eleven SSNs were not classified.

### Predictive factors for SSN growth and for the time to SSN growth

Multivariate analysis was performed with a logistic regression model to predict the incidence of SSN growth after long-term follow-up [7, 10, 20, 21, 24, 25] (Table 3). An initially large SSN size was found to be a risk factor affecting the incidence of SSN growth in 5/6 studies [7, 10, 20, 24, 25]. The other risk factors for the incidence of SSN growth varied among studies, such as age ≥ 65 years and the presence of a solid portion (mGGN) [7, 20] (Table 3). Multivariate Cox proportional hazards regression analysis was conducted to predict the time to SSN growth [3, 10, 21, 23, 24, 26] (Table 4). We also found that the size of the SSN was the most frequent risk factor for the time to SSN growth in 5/6 studies [10, 21, 23, 24, 26] (Table 4).

### Risk of bias assessment

After assessing the studies with the Newcastle–Ottawa scale, 14 of the 16 studies (87.5%) were scored as 4, one (6.25%) was scored as 5, and one (6.25%) was scored as 6 (Supplementary Materials, Table E3). All the studies’ quality was assessed as moderate quality level. There was some publication bias by means of Egger’s test (*p* < 0.001).

## Discussion

The management of persistent SSNs is a topic of importance because an increasing number of SSNs are being identified on chest CT [3, 27]. Clinically, follow-up CT is preferred over immediate surgery because of the indolent behavior, slower growth rate, and good prognosis of SSNs, even if they are malignant [28–31]. Long-term follow-up after the first CT is necessary to accurately assess SSN growth. Because the long-term course of SSNs remains unclear and larger sample studies with long-term follow-up CT are lacking, we performed a systematic review and meta-analysis focused on SSNs with at least 2 years of follow-up. The overall incidence of SSN growth was 22% (95% CI, 15–29%), while the incidence of growth was only 5% (95% CI: 3–7%) after at least 2 years of stability, but both had high heterogeneity. In the studies, we excluded studies in which all patients had a history of malignant tumors, such as breast cancer [32] or had previously undergone surgical treatment of the dominant lung cancer [33, 34]. The natural course of these residual SSNs or incidentally detected SSNs after surgery may be different from other SSNs, and these SSNs also have different follow-up strategies [35].

In our study, the high heterogeneity in the incidence of growth may be caused by many factors, such as different inclusion criteria, definitions of growth, and initial sizes of the SSNs. The initial diameters of the SSNs in the included studies were varied from smaller than 5 to 20 mm [11, 17, 22, 26]. The definition of SSN growth also differed among the studies. The new occurrence of solid parts was not defined as growth in five studies [11, 17–20], while it was defined as growth in the majority of studies even if the size decreased [3, 7, 9, 10, 21, 23–26, 36]. Accurate measurements of SSNs are important to assess their growth and the recommendations from the Fleischner Society addressed pulmonary nodule measurements on CT in 2017 [37]. It was recommended that the average long- and short-axis diameters be used to measure nodule size and a 2-mm threshold should be defined as nodule growth [37–41]. Because SSNs are three-dimensional lesions, an increase in volume or mass could more reliably reflect the growth of SSNs. An increase of at least 20–30% in volume or mass was also used to define SSN growth [22–24]. In addition, to assess the growth of SSNs accurately, we should also consider other morphological changes, such as shape, borders, and internal texture [37]. With the development of advanced semiautomated and automated measurement techniques, the assessment of SSN growth may become more consistent and accurate in the future [42–44].

Additionally, we focused on the incidence of SSN growth after 2 years or more of stability. However, Lee et al [3] reported that 13% of SSNs (27/208) had growth even after 5 years of stability, which is a higher rate than the pooled incidence of growth in the other studies (13% vs. 5%). The possible reasons for this high heterogeneity may be as follows. First, SSNs managed with long-term follow-up are likely to be smaller. Larger persistent SSNs or mGGNs with solid components ≥ 5 mm are more likely to be removed through surgery or other therapies. Some studies [45, 46] have reported that an SSN lesion measuring ≥ 10 mm is a risk factor for invasive adenocarcinomas. The size of SSNs on the initial follow-up CT may be one of the factors that influence the incidence of growth. For example, the initial diameter of the SSNs was smaller than 5 mm in the study by Lee et al [3], but the diameters in the other studies were larger than or equal to 5 mm [7–10, 17, 19, 20, 22]. Therefore, we conducted a subgroup analysis for the SSNs with an initial diameter of ≥ 5 mm and < 5 mm (Fig. 3b).

In these different subgroups, the factors affecting the incidence of growth and the time to growth were analyzed. We found that the size of the SSNs was the factor most frequently associated with growth and the time to growth [7, 10, 20, 21, 23–26]. Therefore, the guidelines state that SSNs can be reasonably and conveniently managed clinically according to their size [1, 2]. Among all 329 SSNs confirmed by pathology, only 4 of 329 (1.2%) were benign, and 307 of 329 (93.3%) were lung adenocarcinomas or precursor glandular lesions. Indeed, SSNs are considered a common form and an indolent subtype of lung adenocarcinoma.

Our study has some limitations. First, the heterogeneity of SSNs in the included studies was high, even in the subgroup analysis of pGGNs. Hence, we further conducted a subgroup analysis of SSNs with at least 2 years of stability and then conducted another subgroup analysis of the initial mean/median diameter ≥ 5 mm. Second, the quality of most included studies was limited according to the Newcastle–Ottawa scale. Finally, some publication bias cannot be ignored. SSNs with a larger size or irregular morphology might be more likely to be treated with aggressive measures such as surgery rather than follow-up. Therefore, most of the analyzed SSNs in the study were smaller than 10 mm, which might have led to some bias.

In conclusion, this systematic review and meta-analysis showed that long-term follow-up CT for SSNs is important and necessary. The overall incidence of growth among SSNs was 22% after a follow-up of 2 years or more. In addition, the pooled incidence of SSN growth after at least 2 years of stability was only 5%. It is anticipated that the present guidelines may serve to standardize our current management of SSNs, but further clarification of their natural history is needed for more precise management.

## Supplementary information

ESM 1 (DOCX 33 kb)

## Abbreviations

95% CI: 95% confidence interval
mGGN: Mixed ground-glass nodule
pGGN: Pure ground-glass nodule
SSN: Subsolid nodule

## Declarations

### Guarantor

The scientific guarantor of this publication is Maosheng Xu.

### Statistics and biometry

No complex statistical methods were necessary for this paper.

### Methodology

• retrospective

• Systematic review

• meta-analysis
