# Supplementary material for: The long-term course of subsolid nodules and predictors of interval growth on chest CT: a systematic review and meta-analysis
Source: Eur Radiol. 2022 Sep 22;33(3):2075–88. doi: 10.1007/s00330-022-09138-y (PMC9935651; doi:10.1007/s00330-022-09138-y)
Supplement: Supplementary file 1 — (DOCX 33 kb) [file 330_2022_9138_MOESM1_ESM.docx]

**Search Strategy**

1. **PubMed search strategy (n=1571):**

#1 "Solitary Pulmonary Nodule"[MeSH Terms] OR "pulmonary nodule*"[Title/Abstract] OR "lung nodule*"[Title/Abstract] OR "ground glass nodule*"[Title/Abstract] OR "ground glass nodule*"[Title/Abstract] OR "ground glass lung nodule*"[Title/Abstract] OR "ground glass pulmonary nodule*"[Title/Abstract] OR "subsolid nodule*"[Title/Abstract] OR "part solid nodule*"[Title/Abstract] OR "partially solid nodule*"[Title/Abstract] OR "partly solid nodule*"[Title/Abstract] OR "non solid nodule*"[Title/Abstract] OR "nonsolid nodule*"[Title/Abstract]

#2 "growth"[Title/Abstract] OR "interval growth"[Title/Abstract] OR "natural growth"[Title/Abstract] OR "tumor growth"[Title/Abstract] OR "tumour growth"[Title/Abstract] OR "growth rate*"[Title/Abstract] OR "nature course"[Title/Abstract] OR "natural history"[Title/Abstract] OR "natural course"[Title/Abstract] OR "nature history"[Title/Abstract] OR "follow-up"[Title/Abstract] OR "follow-up"[Title/Abstract]

#3 "tomography, x ray computed"[MeSH Terms] OR "tomography scanners, x ray computed"[MeSH Terms] OR "computed tomography"[Title/Abstract] OR "computer assisted tomography"[Title/Abstract] OR "CT"[Title/Abstract] OR "CAT"[Title/Abstract]

#4 #1 AND #2

#5 #4 AND #3

1. **Cochrane search strategy (n=116):**

ID Search Hits

#1 MeSH descriptor: [Solitary Pulmonary Nodule] explode all trees 83

#2 ("pulmonary nodule*"):ti,ab,kw 149

#3 ("lung nodule*"):ti,ab,kw 294

#4 ("ground glass nodule*"):ti,ab,kw 12

#5 ("ground-glass nodule*"):ti,ab,kw 12

#6 ("ground glass lung nodule*"):ti,ab,kw 0

#7 ("ground glass pulmonary nodule*"):ti,ab,kw 0

#8 ("subsolid nodule*"):ti,ab,kw 9

#9 ("part-solid nodule*"):ti,ab,kw 6

#10 ("partially solid nodule*"):ti,ab,kw 1

#11 ("partly solid nodule*"):ti,ab,kw 0

#12 ("non-solid nodule*"):ti,ab,kw 1

#13 ("nonsolid nodule*"):ti,ab,kw 1

#14 #1 OR #2 OR #3 OR #4 OR #5 OR #6 OR #7 OR #8 OR #9 OR #10 OR #11 OR #12 OR #13 403

#15 ("growth"):ti,ab,kw 49608

#16 ("interval growth"):ti,ab,kw 11

#17 ("natural growth"):ti,ab,kw 15

#18 ("tumor growth"):ti,ab,kw 1915

#19 ("tumour growth"):ti,ab,kw 1915

#20 ("growth rate*"):ti,ab,kw 1134

#21 ("nature course"):ti,ab,kw 1

#22 ("nature history"):ti,ab,kw 3

#23 ("natural course"):ti,ab,kw 602

#24 ("natural history"):ti,ab,kw 2268

#25 ("follow up"):ti,ab,kw 260672

#26 ("follow-up"):ti,ab,kw 260672

#27 #15 OR #16 OR #17 OR #18 OR #19 OR #20 OR #21 OR #22 OR #23 OR #24 OR #25 OR #26 303046

#28 MeSH descriptor: [Tomography, X-Ray Computed] explode all trees 5276

#29 MeSH descriptor: [Tomography Scanners, X-Ray Computed] explode all trees 41

#30 ("computed tomography"):ti,ab,kw 13957

#31 ("computer assisted tomography"):ti,ab,kw 6206

#32 ("CT"):ti,ab,kw 80365

#33 ("Tomography"):ti,ab,kw 29297

#34 #28 OR #29 OR #30 OR #31 OR #32 OR #33 98313

#35 #14 AND #27 AND #34 116

1. **Web of Science Core Collection search strategy (n=1640):**

#1 TS=("pulmonary nodule*") OR TS=("lung nodule*") OR TS=("ground glass nodule*") OR TS=("ground glass lung nodule*") OR TS=("ground glass pulmonary nodule*") OR TS=("subsolid nodule*") OR TS=("part-solid nodule*") OR TS=("partially solid nodule*") OR TS=("partly solid nodule*") OR TS=("non-solid nodule*")

#2 TS=("growth") OR TS=("growth rate") OR TS=("interval growth") OR TS=("tumor growth") OR TS=("tumour growth") OR TS=("Nature course") OR TS=("Natural history") OR TS=("natural course") OR TS=("Nature history") OR TS=("follow up")

#3 (((TS=("computed tomography")) OR TS=("computed tomography scanner")) OR TS=("CT")) OR TS=("CAT")

#4 ((#1) AND #2) AND #3

1. **Embase search strategy(n=3475):**

No.Query Results Results Date

#14. #9 AND #13 3475 8 Nov 2021

#13. #10 OR #11 OR #12 881526 8 Nov 2021

#12. 'computed tomography':ti,ab,kw OR 'computer 845452 8 Nov 2021

assisted tomography':ti,ab,kw OR 'ct':ti,ab,kw

#11. 'computed tomography scanner'/exp 51392 8 Nov 2021

#10. 'x-ray computed tomography'/exp 76658 8 Nov 2021

#9. #5 AND #8 5105 8 Nov 2021

#8. #6 OR #7 3211447 8 Nov 2021

#7. 'growth rate':ti,ab,kw OR 'interval 1958858 8 Nov 2021

growth':ti,ab,kw OR 'tumor growth':ti,ab,kw OR

'tumour growth':ti,ab,kw OR 'nature

course':ti,ab,kw OR 'natural history':ti,ab,kw OR

'natural course':ti,ab,kw OR 'nature

history':ti,ab,kw OR 'follow up':ti,ab,kw

#6. 'growth'/exp 1353712 8 Nov 2021

#5. #1 OR #2 OR #3 OR #4 28584 8 Nov 2021

#4. 'ground glass nodule*':ti,ab,kw OR 'ground glass 1374 8 Nov 2021

lung nodule*':ti,ab,kw OR 'ground glass pulmonary

nodule*':ti,ab,kw OR 'subsolid nodule*':ti,ab,kw

OR 'part-solid nodule*':ti,ab,kw OR 'partially

solid nodule*':ti,ab,kw OR 'partly solid

nodule*':ti,ab,kw OR 'non-solid nodule*':ti,ab,kw

#3. 'ground glass opacity'/exp 3474 8 Nov 2021

#2. 'peripheral lung lesion'/exp 1237 8 Nov 2021

#1. 'lung nodule'/exp 23874 8 Nov 2021

Table E1. The detail criteria of term for the SSN follow up in included studies.

| **Study** | **SSNs in included studies** |
| --- | --- |
| Takahashi et al, 2012 (17) | 2-year follow-up, and within at least 6 months after the 2-year follow-up |
| Chang et al, 2013 (18) | ＞ 2 years follow up |
| Kobayashi et al, 2013 (19) | ≥ 6-month follow up but recorded the numbers of SSN growth after ≥2 years of stability |
| Lee et al, 2013 (20) | ＞ 2 years follow up |
| Eguchi et al, 2014 (21) | ＞ 2 years follow up |
| SHIN et al, 2014 (22) | ≥ 5 years follow up or diagnosed of cancer within 5-year period |
| Kakinuma et al, 2015(11) | ≥ 5 years follow up or resected within 5 years because of growth or incident newly cases of lung cancer |
| Cho et al, 2016(7) | ＞ 3 years stability |
| Sawada et al, 2016(8) | not mention follow up term but recorded the numbers of SSN growth after ≥2 years of stability |
| SATO et al, 2017(10) | ＞ 2 years follow up |
| Lee et al, 2019 (3) | follow up for 5 years stablility |
| Qi et al, 2019 (23) | follow-up of ≥ 2 years and those with a follow-up of < 2 years but that had grown |
| Shi et al, 2019 (24) | ＞ 2 years follow up |
| Gao et al, 2020 (25) | follow-up ≥ 2 years |
| Qiu et al, 2020 (26) | ≥ 3 years follow up |
| Lee et al, 2020 (9) | follow up after 5 years of stability |

Table E2. The subgroup analysis of the initial mean/median diameter ≥ 5 mm and < 5 mm for subsolid nodules after two years of stability or more.

| **Study** | **Initial mean/ median diameter（mm）** | **Initial mean/ median diameter**（mm） |
| --- | --- | --- |
| Takahashi, et al 2012 (17) | 8.1 ± 2.4 | diameter ≥5mm |
| Kobayashi et al, 2013 (19) | 9.5 (4 - 25) | diameter ≥5mm |
| Lee et al, 2013 (20) | 7.8 ± 4.4 | diameter ≥5mm |
| SHINet al, 2014 (22) | 7 ± 1.2 | diameter ≥5mm |
| Choet al, 2016(7) | 5.0 (2.0 - 31.1) | diameter ≥5mm |
| Sawada et al, 2016(8) | 10 (3 - 30) | diameter ≥5mm |
| SATO et al, 2017(10) | 12.2 ± 6.1 | diameter ≥5mm |
| Lee et al, 2019 (3) | 4.33 (2.67–5.75) | diameter ＜5mm |
| Lee et al, 2020 (9) | 9.33(7-17.33) | diameter ≥5mm |

Table E3. According to the Newcastle-Ottawa scale, study quality was assessed for the included studies.

| **ID** | **Study Name and Year** | **Case Definition** | **Cases Representativeness** | **Controls Selection** | **Controls Definition** | **Study Controls** | **Study Controls for any Additional Factor** | **Exposure Ascertainment** | **Ascertainment Method** | **Non-Response Rate** | **Total** |
| --- | --- | --- | --- | --- | --- | --- | --- | --- | --- | --- | --- |
| 1 | Takahashi 2012 | 1 | 1 | 0 | 1 | 0 | 0 | 0 | 1 | 0 | 4 |
| 2 | Chang 2013 | 1 | 1 | 0 | 1 | 0 | 0 | 0 | 1 | 0 | 4 |
| 3 | Kobayashi 2013 | 1 | 1 | 0 | 1 | 0 | 0 | 0 | 1 | 0 | 4 |
| 4 | Lee 2013 | 1 | 1 | 0 | 1 | 0 | 0 | 0 | 1 | 0 | 4 |
| 5 | Eguchi 2014 | 1 | 1 | 0 | 1 | 0 | 0 | 0 | 1 | 0 | 4 |
| 6 | SHIN 2014 | 1 | 1 | 1 | 1 | 0 | 0 | 0 | 1 | 0 | 5 |
| 7 | Kakinuma 2015 | 1 | 1 | 1 | 1 | 0 | 0 | 1 | 1 | 0 | 6 |
| 8 | Cho 2016 | 1 | 1 | 0 | 1 | 0 | 0 | 0 | 1 | 0 | 4 |
| 9 | Sawada 2016 | 1 | 1 | 0 | 1 | 0 | 0 | 0 | 1 | 0 | 4 |
| 10 | SATO 2017 | 1 | 1 | 0 | 1 | 0 | 0 | 0 | 1 | 0 | 4 |
| 11 | Lee 2019 | 1 | 1 | 0 | 1 | 0 | 0 | 0 | 1 | 0 | 4 |
| 12 | Qi 2019 | 1 | 1 | 0 | 1 | 0 | 0 | 0 | 1 | 0 | 4 |
| 13 | Shi 2019 | 1 | 1 | 0 | 1 | 0 | 0 | 0 | 1 | 0 | 4 |
| 14 | Gao 2020 | 1 | 1 | 0 | 1 | 0 | 0 | 0 | 1 | 0 | 4 |
| 15 | Qiu 2020 | 1 | 1 | 0 | 1 | 0 | 0 | 0 | 1 | 0 | 4 |
| 16 | Lee 2020 | 1 | 1 | 0 | 1 | 0 | 0 | 0 | 1 | 0 | 4 |
